# Reviews of Dental Literature

**Published:** 1904-11

**Authors:** 


### Reviews of Dental Literature.

Medical Education in the United States.—The present issue of the *Journal* is the fourth annual educational number, and contains statistics regarding medical education in the United States, covering the year ending June 30, 1904. The information contained in this statistical study was obtained largely from the colleges directly, and has been certified to by some one in authority in each school, so that we have every reason to believe that all the data are as correct as can be obtained. It has been no easy task to gather all this information, and any errors that may have crept into the work are only such as are likely to occur when information is gathered from many sources. Only one college in the United States (not including the Manila and San Juan schools) flatly refused to give any information, but the data which might have been obtained would not have changed the totals appreciably. We take this opportunity of extending our thanks to all those who aided us in gathering these statistics.

A perusal of our study will show that medical education, so far as students and colleges are concerned, has not changed materially during the past year, although a slight improvement is noticeable in the advances made in the length of the college term. This improvement is, on the whole, very gratifying, inasmuch as it shows the disposition of all the schools to better medical education. In the statistics contained in this number, we have embodied some new features which, we are convinced, will be of interest to medical educators.

*Number of Medical Students.*—The number of medical students in the United States for the year ending June 30, 1904, was 26,138, a decrease of 1477 below the year 1903. Of this number, 23,662 were in attendance at the regular schools; 1105 at the homœopathic; 1014 at the eclectic, and 357 at the physiomedical and nondescript schools. There was a decrease in the attendance of the regular schools of 1268 below last year, and a decrease of 1216 below the year previous,—1902. In the homœopathic schools there was a decrease of 393 below that of 1903, and a decrease of 512 below 1902. The eclectic schools have been increasing steadily since

1900. In 1904, 1014 students attended the eclectic schools, an increase of 166 over the attendance of the year previous,—1903. The physiomedical and nondescript schools show an increase in attendance of 18 over the previous year, the attendance in 1903–04 being 357. This increase, however, occurred in the nondescript schools and not in the physiomedical.

TABLE OF MEDICAL COLLEGE ATTENDANCE.

| Year. | Reg. | Homœo. | Eclec. | Physiomed. and Nondes. | Total. |
|---|---|---|---|---|---|
| 1880 | 9,776 | 1220 | 830 | ... | 11,826 |
| 1890 | 13,521 | 1164 | 719 | ... | 15,404 |
| 1900 | 22,710 | 1909 | 552 | ... | 25,171 |
| 1901 | 23,846 | 1683 | 664 | 224 | 26,417 |
| 1902 | 24,878 | 1617 | 765 | 241 | 27,501 |
| 1903 | 24,930 | 1498 | 848 | 339 | 27,615 |
| 1904 | 23,662 | 1105 | 1014 | 357 | 26,138 |

*Number of Graduates.*—The total number of graduates for the year ending June 30, 1904, was 5747, an increase of 49 over the preceding year. The increase in 1903 over 1902 was 699, so that the increase during the present year was much less than that of the year previous. Of course, there are nine more colleges this year than there were last year, but three of the nine were not in session, and the others, with the exception of one, taught only a portion of the medical course. Although the graduates have increased slightly, the matriculants have decreased considerably, and we must assume that the decrease has occurred largely in the Freshmen classes, partly because of the increase in entrance requirements, partly because of the increase in fees and general expense of the medical course, and, perhaps, because of the prosperity in the business world in general, which usually lowers the attendance in the professional schools. In some colleges there was a decided falling off in the Freshmen class, while in others there was a very slight increase. The falling off was noticeable, particularly, in those schools that raised their entrance requirements. The decrease in the number of graduates in the homœopathic schools—49—represents the lowest number of graduates since 1902. The eclectic schools show a decrease of 3 in the number of graduates below last year, and the other school a decrease of 1. In the regular schools, on the other hand, there has been an increase of 102 over 1903.

TABLE OF MEDICAL COLLEGE GRADUATES.

| Year. | Reg. | Homœo. | Eclec. | Physiomed. and Nondes. | Total. |
|---|---|---|---|---|---|
| 1880 | 2673 | 380 | 188 | .. | 3241 |
| 1890 | 3853 | 380 | 221 | .. | 4454 |
| 1900 | 4715 | 413 | 86 | .. | 5214 |
| 1901 | 4879 | 387 | 148 | 30 | 5444 |
| 1902 | 4498 | 336 | 138 | 27 | 4999 |
| 1903 | 5088 | 420 | 149 | 41 | 5698 |
| 1904 | 5190 | 371 | 146 | 40 | 5747 |

*Number of Colleges.*—Our report last year showed that there were at that time one hundred and fifty-seven medical colleges, three of which did not grant the degree of M.D., but taught only the first two years of the medical curriculum. Since then one college has passed out of existence, and ten new ones have been formed, making a total of one hundred and sixty-six colleges at the present time. Of these one hundred and thirty-three are regular, nineteen homœopathic, ten eclectic, three physiomedical, and one institution which teaches all the "pathies" and "isms," including osteopathy. Of the regular colleges, two are not yet active, and seven do not grant any degree. Of the latter number, six teach only the first two years of the medical course, and one only the first year. Two of the regular colleges are located in our island possessions; one is the Medical Department of the University of Porto Rico at San Juan, and the other the Medical Department of the San Tomaso University of Manila, P. I.

COLLEGES IN STATES AND CITIES.

ALABAMA—2.

Birmingham ........ 1

Mobile ........ 1

ARKANSAS—1.

Little Rock ........ 1

CALIFORNIA—8.

San Francisco ........ 5

Oakland ........ 1

Los Angeles ........ 2

COLORADO—3.

Denver ........ 2

Boulder ........ 1

CONNECTICUT—1.

New Haven ........ 1

DISTRICT OF COLUMBIA—3.

Washington ........ 3

GEORGIA—3.

Augusta ........ 1

Atlanta ........ 2

ILLINOIS—16.

Chicago ........15

Galesburg ........ 1

INDIANA—6.

Indianapolis ........ 4

Fort Wayne ........ 1

Bloomington ........ 1

| Iowa—5. | |
|---|---|
| Iowa City | 2 |
| Des Moines | 1 |
| Sioux City | 1 |
| Keokuk | 1 |

| Kansas—3. | |
|---|---|
| Lawrence | 1 |
| Topeka | 1 |
| Kansas City | 1 |

| Kentucky—7. | |
|---|---|
| Louisville | 7 |

| Louisiana—2. | |
|---|---|
| New Orleans | 2 |

| Maine—1. | |
|---|---|
| Portland | 1 |

| Maryland—8. | |
|---|---|
| Baltimore | 8 |

| Massachusetts—4. | |
|---|---|
| Boston | 4 |

| Michigan—6. | |
|---|---|
| Ann Arbor | 2 |
| Detroit | 3 |
| Grand Rapids | 1 |

| Minnesota—3. | |
|---|---|
| Minneapolis | 3 |

| Mississippi—1. | |
|---|---|
| Oxford | 1 |

| Missouri—15. | |
|---|---|
| St. Louis | 6 |
| Kansas City | 6 |
| Columbia | 1 |
| St. Joseph | 2 |

| Nebraska—3. | |
|---|---|
| Omaha | 2 |
| Lincoln | 1 |

| New Hampshire—1. | |
|---|---|
| Hanover | 1 |

| New York—11. | |
|---|---|
| New York City | 8 |
| Albany | 1 |
| Buffalo | 1 |
| Syracuse | 1 |

| North Carolina—4. | |
|---|---|
| Raleigh | 2 |
| Davidson | 1 |
| Wake Forest | 1 |

| Ohio—10. | |
|---|---|
| Cincinnati | 4 |
| Cleveland | 3 |
| Columbus | 2 |
| Toledo | 1 |

| Oklahoma—1. | |
|---|---|
| Norman | 1 |

| Oregon—2. | |
|---|---|
| Salem | 1 |
| Portland | 1 |

| Pennsylvania—7. | |
|---|---|
| Philadelphia | 6 |
| Pittsburg | 1 |

| Philippine Islands—1. | |
|---|---|
| Manila | 1 |

| Porto Rico—1. | |
|---|---|
| San Juan | 1 |

| South Carolina—1. | |
|---|---|
| Charleston | 1 |

| Tennessee—11. | |
|---|---|
| Nashville | 4 |
| Knoxville | 2 |
| Chattanooga | 2 |
| Memphis | 1 |
| Jackson | 1 |
| Sewanee | 1 |

| | |
|---|---|
| **Texas—8.** | |
| Galveston | 1 |
| Fort Worth | 1 |
| Dallas | 5 |
| Texarkana | 1 |
| **Vermont—1.** | |
| Burlington | 1 |
| **Virginia—3.** | |
| Charlottesville | 1 |
| Richmond | 2 |
| **West Virginia—1.** | |
| Morgantown | 1 |
| **Wisconsin—2.** | |
| Milwaukee | 2 |

Three colleges are exclusively for women; sixty for men; one hundred and three are co-educational; four hold only night sessions, and two both day and night sessions. There are seven schools to which only colored people are admitted. Four schools operate under the continuous course system, the year being divided into quarters, the student being allowed to attend only a specified number of quarters or semesters in each calendar year. Sixty-six regular schools, four homœopathic, and one eclectic college have a university connection or affiliation. The baccalaureate and medical degrees are granted at the end of six years' study by six colleges, and at the end of seven years by one college.

Seventy regular colleges are members of the Association of American Medical Colleges, twelve belong to the Southern Medical College Association, eighteen of the homœopathic schools are recognized as in good standing by the American Institute of Homœopathy, and eight of the eclectic colleges are members of the National Confederation of Eclectic Medical Colleges. Many of the colleges not in these associations abide by their entrance requirements.

A study of the following comparative table of medical colleges is of interest. The regular schools have increased in number since 1903, while the other medical colleges number as many as last year. It must be remembered, however, that last year only three schools gave instruction in the first two years' work of the medical curriculum, whereas this year seven schools were engaged in doing this preparatory work. Each of these preparatory schools are integral parts of recognized universities, and this work, therefore, is accepted as a full credit by other medical colleges. By subtracting these seven colleges, and also the two schools in Porto Rico and Manila, from the number of regular medical schools, it gives us an actual increase of only two colleges which grant the degree of M.D., or a total of one hundred and fifty-six.

COMPARATIVE TABLE OF MEDICAL COLLEGES.

| Year. | Reg. | Homœo. | Eclec. | Physiomed. and Nondes. | Total. |
|---|---|---|---|---|---|
| 1880 | 72 | 12 | 6 | .. | 90 |
| 1890 | 93 | 14 | 9 | .. | 116 |
| 1900 | 121 | 22 | 8 | .. | 151 |
| 1901 | 124 | 21 | 10 | 4 | 159 |
| 1902 | 121 | 20 | 10 | 4 | 155 |
| 1903 | 121 | 19 | 10 | 4 | 154 |
| 1904 | 133 | 19 | 10 | 4 | 166 |

*Length of Terms.*—A study of the length of terms, in months, of the various medical colleges discloses some very interesting facts. Of the one hundred and sixty-three schools from which we were able to obtain the necessary information, 40.3 per cent. have a course of at least eight months' duration. Only 16.3 per cent. have a course of less than seven months' duration. The figures are as follows:

TABLE OF COLLEGE TERMS.

| Term. | Schools. | Per cent. |
|---|---|---|
| 6 months | 27 | 16.3 |
| 7 months | 44 | 27.0 |
| 7½ months | 22 | 13.5 |
| 8 months | 34 | 20.8 |
| 8½ months | 13 | 7.9 |
| 9 months | 19 | 11.6 |
| 10 months * | 4 | 2.4 |

* Night Schools.

Nearly all the shorter term schools are located in the South, where medical educators feel that the conditions are such as to prohibit a longer term. Most of the non-sectarian schools have seven months' terms. A very small percentage of the regular colleges have less than a seven months' term. Many of the colleges which last year had seven months' terms have adopted the eight and nine months' terms for the coming year. It is probable that another year will see the passing of the six months' school. Of course, the term "months" is elastic, inasmuch as seven months means anywhere from twenty-six to twenty-eight weeks; eight months, thirty to thirty-two weeks; and nine months, thirty-three to thirty-six weeks. It would be far better to regulate the length of each annual course by specifying a definite number of teaching days or number of hours spent in college.

*Women in Medicine.*—It is of interest that, in spite of the apparent passing away of colleges for women, the number of women medical students and graduates has been increasing steadily. During the past year only two of the three colleges for women were in session, but ninety-seven colleges are co-educational, which may account for the increase in women students. During the past year 1129 women were engaged in the study of medicine—4.3 per cent. of the total number of medical students—and 244 graduated,—four per cent. of the total number of graduates. Of the total number of matriculants, only 183 were in attendance at the two woman's colleges, and 46 graduated from them.—Editorial, *Jour. Amer. Med. Assn.*

The Teaching of Materia Medica in Medical Schools. Abstract of a paper by Torald Sollman, M.D.[1]

DESIRABILITY OF RESTRICTING THE COURSE OF MATERIA MEDICA.

"I must confess, if I had my way I should abolish materia medica[2] altogether. . . . Not one trace of a knowledge of drugs has remained in my memory from that time to this; and really, as a matter of common sense, I cannot understand the arguments for obliging a medical man to know all about drugs, and where they come from. Why not make him belong to the Iron and Steel Institute, and learn something about cutlery, because he uses knives?

"I entertain a very strong conviction that any one who adds to medical education one iota or tittle beyond what is absolutely necessary is guilty of a very grave offence."—Huxley, "On Medical Education," 1870.

Every one will agree with this last sentence. In most medical schools the required class attendance exceeds thirty hours per week, leaving the student a very insufficient time for home study. This condition tends naturally to become worse, for the domain of medical knowledge is constantly extending, and the teaching must follow these extensions. It is, therefore, imperative to lighten the curriculum as much as possible by curtailing such studies as are no longer essential.

A case in point is presented by the relation of pharmacology, materia medica, and therapeutics. The study of the action of drugs

[1] Professor of Pharmacology and Materia Medica, Western Reserve University, Cleveland, Ohio.

[2] It will, I hope, be understood that I do not include therapeutics under this head.

by laboratory methods is a new branch and one which requires considerable time, which must be taken from some other subjects. It is very true that a knowledge of pharmacology facilitates the study of therapeutics so greatly that a part of the time formerly devoted to therapeutics can be assigned to pharmacology without injury. The saving thus effected is not, however, sufficient. What further time is needed can be profitably drawn from materia medica (including pharmacy) if the course in these branches is wisely reorganized. This can be readily done, especially if they are taught in the same department as pharmacology, as they should be, in my opinion.

No one can doubt that the action of drugs is of vastly greater importance to the medical practitioner than their natural history. The latter can only be of essential importance when the physician is located remote from a pharmacist, a condition which is rather rare in this country. Nothing prevents those who expect to be so situated from taking a special, optional course, and such should be offered by the college, if it is needed. Were there no other demand on the students' time, an exhaustive course of materia medica might be profitable to all other students, but there can be no question that the other demands are more important. Indeed, I incline to the belief that the above advice of Huxley has not been sufficiently heeded by many schools, doubtless because it is somewhat too radical.

### WHAT PARTS OF MATERIA MEDICA SHOULD BE RETAINED?

How far, then, may the study of materia medica be safely curtailed? What is essential? What may be made optional and what omitted entirely? There is surely room for much honest difference of opinion in this connection. I venture to give my views, as they may serve as a basis for reflection.

I believe that the students should be given, by way of introduction, some general ideas about the classes of chemical and structural constituents of drugs. Sufficient botany should have been learned in school, and zoölogy is quite superfluous. The systems of weights and measures should be made thoroughly familiar. In regard to pharmacy, the student should learn the different classes of pharmaceutic preparations, their common characteristics and special uses, and he should be given (by demonstration) a broad conception of how they are prepared. A little practice in simple dispensing is very desirable. All these data will be invaluable in understanding the materia medica of special drugs.

As regards the latter, only those should be discussed at all which have a real, living therapeutic or toxicologic importance. Of the former the student should be required to know, in the first place, those data which are needed in prescribing: The correct Latin name (faulty orthography indicates a faulty education, in materia medica as elsewhere), the methods of administration, the most useful preparations, *and these only!* [1] Their dose, solubility, incompatibility, and in a few cases, their composition; constituents should be taught only in so far as important therapeutically or by incompatibility.

Furthermore, I believe that there should be required considerable familiarity with the appearance and other physical characters of the important drugs and their principal preparations, so that the more common poisons may be identified or excluded. This seems to me very important, and I believe that I lay rather more stress on it than is commonly done. The knowledge so obtained is also very valuable, in that it often enables the physician to adapt the medicine to the peculiarities of the patient, and sometimes to prevent the results of an error on the part of a druggist. I require this acquaintance rather oftener in the case of preparations than of crude drugs. I need hardly add that a good deal of discrimination must be exercised in selecting the drugs to be studied. The study must, of course, be made directly from the specimen.

It will be seen that the principal abridgment which I advocate in the teaching of materia medica concerns the number of drugs and preparations to be studied, neglecting all those—and they are quite numerous—which are unimportant.[2] A further saving is obtained by limiting pharmacy, and by paying no attention to habitat, natural order, method of collection, etc. These are quite useless for practical purposes, and those students who are interested

---

[1] There is some excuse for the Pharmacopœia retaining obsolete drugs and preparations, because these are sometimes used in certain localities or by the laity, but there is no reason for burdening medical students with them. It is to be wished that all State boards would discourage such questions as, "Give *all* the preparations of ——."

[2] This refers only to materia medica. It is often desirable to say a few words about the action of these unimportant drugs in the lectures on pharmacology.

in them can easily find the information. The natural orders are sometimes useful in explaining relationships, but these can be touched on in pharmacology. In this way, and by arranging the course as indicated below, the class-work in materia medica and pharmacy may be reduced to something like forty-five hours. Besides this required work, I believe that a more thorough study of the chemistry of drugs and of pharmacy is very useful, not so much by the direct knowledge which they give, but because the handling of drugs which they require gives a greater familiarity. This advantage is not sufficient, to my mind, to make them compulsory, but they may be offered as elective or optional work.

### ARRANGEMENT OF THE INSTRUCTION.

The dryness of materia medica is almost proverbial, and it is, indeed, difficult to render interesting a study which consists so largely of memorizing. This difficulty is enhanced by constituting the subject into a separate course, as is so often done, and especially by letting this course precede that of pharmacology or therapeutics. What interest can there be for the student in memorizing certain tabulated information which he does not fully understand, about a drug which, too often, he has never seen, and about the uses of which he has no clear idea? What opportunities I have had of observing have only confirmed my objections to this plan. I believe that only the preliminary subjects of pharmacy, etc., should be taught separately, and that the materia medica of the individual drugs should be studied immediately *after* their action and uses. It is my practice to give the student his first experience with drugs by letting him use them in the laboratory, as this is the most efficient way of arousing his interest and showing him the importance of the drug. When he has in this way obtained an objective knowledge of the effects of most drugs and has become curious to have them explained, their action is studied systematically in class, and after each lecture the students are supplied with the specimens of drugs, with instructions to describe them, again objectively, in their note-books. Around this nucleus the other information is grouped. In this way the interesting parts of the study carry the more mechanical parts, so that these are less felt. It will be noticed that lectures are not emphasized in this scheme. If there is any study in which formal lectures are misplaced this is certainly materia medica. The subject is taught with us almost purely by laboratory work and recitations.—*Jour. Amer. Med. Assn.*

